# Silencing of interferon regulatory factor gene 6 in melanoma

**DOI:** 10.1371/journal.pone.0184444

**Published:** 2017-09-06

**Authors:** Yoshimasa Nobeyama, Hidemi Nakagawa

**Affiliations:** Department of Dermatology, The Jikei University School of Medicine, Minato-ku, Tokyo, Japan; University of Navarra, SPAIN

## Abstract

**Background:**

Methylation of a CpG island (CGI; a dense cluster of CpGs) located in the 5' region of a gene suppresses transcription of that gene. Interferon regulatory factor 6 (IRF6) is associated with the expression of interferon, which is used as an effective adjuvant therapy for melanoma, and is regarded as a tumor suppressor. However, little is known about the methylation status of the *IRF6* gene in melanoma.

**Objective:**

The purpose was to determine the methylation status of the CGI located in the 5' region of *IRF6* (5' *IRF6* CGI) in melanoma.

**Methods:**

Quantitative real-time methylation-specific PCR (RT-MSP) and bisulfite sequencing were performed to examine *IRF6* gene methylation status. Quantitative real-time reverse transcription-PCR (RT-PCR) was performed to examine *IRF6* expression.

**Results:**

The methylation level of the 5' *IRF6* CGI was completely inversely correlated with cell sensitivity to interferon-β in eight examined melanoma cell lines. These methylation levels were high in the melanoma cell lines with suppression of *IRF6* expression and were low in the cell lines with *IRF6* expression. The methylation levels of the 5' *IRF6* CGI ranged widely from 0.0% to 65.4% in 21 clinical melanoma samples but showed a narrow range of low levels between 0.0% to 7.2% in 24 clinical melanocytic nevus samples. These methylation levels were not associated with clinical parameters except for melanoma subtypes.

**Conclusion:**

*IRF6* is aberrantly silenced by DNA methylation of the 5' *IRF6* CGI in melanoma. The methylation status of *IRF6* is potentially associated with the sensitivity of melanoma to interferon.

## Introduction

DNA methylation is a DNA modification resulting from the covalent binding of a methyl group to a DNA nucleotide such as the cytosine of a CpG dinucleotide, where a 5' cytosine is adjacent to a 3' guanine [1,2]. The methylation status of individual CpG sites is faithfully copied into daughter cells [3]. CpG islands (CGIs) are dense clusters of CpGs that are often located in the 5' regions of genes. Methylation of a CGI located in the 5' region of a gene suppresses transcription of that gene [4]. In normal cells, the CGIs located in the 5' regions of most genes are unmethylated, resulting in the expression of those genes [4]. However, in malignant cells, a number of CGIs located in the 5' regions of genes, including tumor-suppressor genes, may be methylated, resulting in the suppression of the transcription of those genes [4,5].

Melanoma is a malignant tumor that develops through transformation of melanocytes [6]. Advanced melanomas often show dismal outcomes [7,8], although immune checkpoint modulators such as anti-programmed death receptor-1 antibodies and anti-cytotoxic T-lymphocyte-associated protein 4 antibodies as well as molecular-targeted agents such as BRAF inhibitors and MEK inhibitors have been reported to improve the prognosis of patients with advanced melanoma to some degree [9]. Based on these dismal outcomes, adjuvant therapies to enhance the inhibition of recurrence and metastases are still needed.

Interferon (IFN) is in widespread use as an adjuvant therapy, mainly for stage II and resectable stage III melanoma (according to the staging system of the American Joint Committee on Cancer) [10] due to its effective inhibition of melanoma progression. Eggermont et al. reported that high-dose IFN-α-2b and pegylated IFN can improve relapse-free survival in prospective, randomized, multicenter treatment trials [10]. In addition, Yamamoto et al. suggested that local administration of natural-type IFN-β potentially improves prognosis including the 5-year survival rate [11]. Suppression or low expression of specific genes in the interferon pathway is associated with poor prognosis of melanoma [12].

Interferon regulatory factors (IRFs), which include nine members, are associated with tumor suppression and cell differentiation [13,14,15,16,17]. IRFs exert their functions through various molecular mechanisms including: (i) regulation of type 1 IFN expression via toll-like receptor signaling [18]; (ii) interaction with the NF-κB signaling pathway [19]; and (iii) interaction with the mammary serine protease inhibitor (maspin), which is regarded as a tumor suppressor [20]. Among the nine IRF members, the tumor-suppressive functions of IRF6 have been supported by many studies, in addition to its important function in the development process of palatal fusion [21]. Thus, Botti et al. reported that IRF6 exhibits a tumor-suppressive function in squamous cell carcinomas [22]; Restivo et al. reported that IRF6 exerts tumor-suppressive functions in keratinocytes through mediation of Notch pro-differentiation [23]; Bailey et al. reported that IRF6 expression is decreased in invasive breast cancer cell lines and breast tumors [20] and that IRF6 facilitates mammary epithelial cell entry into the G0 phase of the cell cycle in collaboration with maspin [24].

Despite these diligent studies, little is known regarding the regulation of *IRF6* by DNA methylation in melanomas or regarding the association between *IRF6* methylation status and sensitivity to IFN. The present study was conducted to clarify the expression and methylation status of the CGI in the 5' region of *IRF6* (5' *IRF6* CGI) in melanomas.

## Materials and methods

### Ethics statement

The ethics committee of The Jikei University School of Medicine granted approval for this study, and written informed consent for the use of tissue samples was obtained from reachable donors or their legal guardians. The ethics committee of The Jikei University School of Medicine waived the requirement for consent from unreachable donors.

### Cell lines, clinical samples, and extraction of nucleic acid

The human melanoma cell lines A375, Hs294T, SH-4, SK-MEL-2 SK-MEL-5, SK-MEL-28 and RPMI-7951, and two normal human cultured epidermal melanocytes (MC1 and MC2) were purchased from the American Type Culture Collection (Manassas, VA). The melanoma cell line G-361 was purchased from the Riken BioResources Center (Tsukuba, Japan). Twenty-one paraffin-embedded melanoma samples were obtained from patients with melanoma (Table 1). Twenty-four melanocytic nevus samples were obtained from patients without any history of melanoma (Table 2).

**Table 1:** Characteristics of the patients who donated melanoma samples.

ID	Age (yrs)	Sex	TNM classification			Outcome*	Type^ǂ^	M-level (%)^¶^
			T	N	M			
1	45	M	2b	1b	0	Dead, 108.7 months	Acral	4.5
2	70	M	4a	1b	0	Alive, 24.9 months	CSD	0.4
3	59	M	4b	3	0	Alive, 25.4 months	Acral	50.5
4	73	M	4b	0	0	Dead, 57.9 months	Acral	2.5
5	59	M	4b	2b	0	Alive, 25.4 months	Acral	28.8
6	59	M	4b	2b	0	Alive, 24.6 months	Acral	0.2
7	50	M	4a	2a	1a	Alive, 50.6 months	NCSD	0.5
8	76	M	3b	0	0	Alive, 19.3 months	Acral	20.6
9	58	M	3a	0	0	Dead, 30.3 months	NCSD	0.2
10	60	M	X	3	0	Dead, 9.1 months	NCSD	64.2
11	77	M	3b	2c	0	Alive, 1.6 months	Acral	0.9
12	75	M	4b	3	0	Dead, 9.0 months	CSD	0.0
13	72	F	4b	1b	1c	Dead, 16.8 months	CSD	0.0
14	72	F	3a	0	0	Dead, 7.5 months	Acral	0.8
15	46	F	4b	1a	0	Alive, 41.3 months	NCSD	0.1
16	52	M	4a	2a	1c	Alive, 26.9 months	NCSD	0.0
17	82	F	4b	1b	0	Alive, 15.1 months	Acral	65.4
18	77	M	4b	X	1c	Alive, 8.4 months	Acral	30.3
19	63	M	4a	0	0	Dead, 14.1 months	NCSD	0.2
20	49	M	4b	0	0	Alive, 27.9 months	Acral	0.0
21	61	F	4a	1a	0	Alive, 11.6 months	NCSD	0.0

*If the patient had died, the interval from diagnosis to death is stated. If the patient was alive at the end of the study, the period from diagnosis to the last day when the patient was confirmed to be alive is stated.

^ǂ^ The definition of melanoma subtype is accorded by Curtin's classification.

^¶^ Methylation level.

M: male; F: female. Non CSD, melanoma arising from non-chronically sun-damaged skin; CSD, melanoma arising from chronically sun-damaged skin; Mucosal, mucosal melanoma; Acral, acral melanoma.

**Table 2:** Characteristics of the patients who donated melanocytic nevus samples.

ID	Age (yrs)	Sex	Site of occurrence	Methylation level (%)
22	5	M	Trunk	7.2
23	23	M	Lower limb	0.0
24	28	F	Trunk	0.0
25	33	F	Upper limb	0.6
26	36	F	Trunk	2.3
27	18	F	Head/Neck	0.0
28	20	F	Upper limb	0.1
29	6	M	Lower limb	1.8
30	3	F	Trunk	1.9
31	6	M	Upper limb	0.0
32	5	F	Trunk	0.0
33	32	M	Head/Neck	0.0
34	16	F	Trunk	0.0
35	24	F	Trunk	1.9
36	17	F	Trunk	0.0
37	10	F	Trunk	0.4
38	27	F	Upper limb	0.0
39	31	M	Head/Neck	0.9
40	15	F	Trunk	0.1
41	6	M	Trunk	0.4
42	56	M	Head/Neck	0.2
43	22	F	Head/Neck	0.0
44	25	F	Lower limb	0.0
45	31	M	Lower limb	0.1

M: male; F: female.

Melanoma and melanocytic nevus were diagnosed histopathologically by at least two experienced board-certified dermatologists and pathologists. The TNM classification of cutaneous melanoma was evaluated according to the American Joint Committee on Cancer melanoma staging system (7th edition). Melanoma samples were classified into four subtypes consisting of melanoma arising from non-chronically sun-damaged skin (NSCD), melanoma arising from chronically sun-damaged skin (CSD), mucosal melanoma and acral melanoma [25].

To extract DNA from paraffin-embedded samples, the samples were sliced into 4 to 10 μm-thick sections, deparaffinized, and then dissected with a fine needle. Genomic DNA was extracted by using a standard phenol/chloroform extraction and ethanol precipitation procedure or the QIAamp DNA mini kit (Qiagen, Valencia, CA). Total RNA was isolated using ISOGEN (Nippon Gene, Tokyo, Japan).

### Treatment with 5-aza-2'-deoxycytidine

For 5-aza-2'-deoxycytidine (5-aza-dC) treatment, melanoma cells were seeded at a density of 1.0×10^5^ to 2.5×10^5^ cells per 10-cm dish on day 0 and were exposed to medium containing 1.0 μM 5-aza-dC (Sigma-Aldrich, St Louis, MO) on days 1 and 3. The cells were harvested on day 5. All cell lines showed mild growth suppression on day 5, when compared with untreated cells.

### Treatment with natural type IFN-β

To obtain cells treated with natural type IFN-β, melanoma cells were seeded at a density of 2.0×10^5^ to 5.0×10^5^ cells per 10-cm dish on day 0 and were exposed to medium containing 1.0×10^3^ international units/ml of natural type IFN-β (Toray Medical, Tokyo, Japan) or phosphate buffered saline (PBS) on day 1. On day 3, the cell numbers were harvested.

To evaluate growth of cells treated with natural type IFN-β, melanoma cells were seeded at a density of 2.0×10^5^ to 5.0×10^5^ cells per 10-cm dish on day 0 and were exposed to medium containing 1.0×10^3^ international units/ml of natural type IFN-β or PBS on days 1 and 3. On day 5, the cell numbers were counted. Growth rate was defined as the value of the number of IFN-β-treated cells divided by the number of PBS-treated cells.

### Quantitative real-time methylation-specific PCR (RT-MSP) and bisulfite sequencing

One μg of the *Bam*HI-digested genomic DNA was modified by sodium bisulfite using the EZ DNA Methylation-Gold Kit (Zymo Research, Irvine, CA) according to the instruction manual, and was dissolved in 40 μl of buffer.

For RT-MSP, 1.0 μl of the sodium bisulfite-treated DNA was amplified with the 7500 Real-Time PCR System (Applied Biosystems, Foster City, CA) using a mixture of primer sets that were specific to the methylated or unmethylated DNA sequence (M or U set, respectively) and the SYBR Green PCR Master Mix I (Toyobo, Osaka, Japan). The sequences of the M set primers were: 5'-GTAGGGTGGGACGTTGGACGGAC-3' for the forward primer and 5'-TAACCACGCCCCCCGACGTTCG-3' for the reverse primer, which were designed to amplify from ‒127 bp to ‒22 bp of the *IRF6* gene based on the major *IRF6* start site (based on the *IRF6* sequence in NC_000001.11). The sequences of the U set primers were: 5'-GGGTTGGGTAGTTTAGAAATGT-3' for the forward primer and 5'-TATAACCACACCCCCCAACA-3' for the reverse primer, which were designed to amplify from ‒98 bp to ‒20 bp of the *IRF6* gene based on the major *IRF6* start site. The number of molecules of a specific sequence in a sample was measured by comparing its amplification with that of standard samples, which contained 10^1^ to 10^8^ copies of the molecule. The methylation level was defined as the number of methylated DNA molecules divided by the total number of methylated and unmethylated DNA molecules. DNA methylated with *Sss*I methylase (New England Biolabs, Beverly, MA) and DNA amplified with the GenomiPhi DNA amplification kit (GE Healthcare Bioscience, Little Chalfont, UK) were used as methylated and unmethylated DNA controls, respectively, under specifically amplified conditions for M and U sets.

For bisulfite sequencing, 1.0 μl of the sodium bisulfite-treated DNA was used for PCR with primers common to methylated and unmethylated DNA sequences. The sequences of these primers were: 5'-GGAGTTAGAAGYGGAGGAGTAG-3' for the forward primer, in which Y indicates a C or a T, and 5'-ACGCCTCCCAAATATAACCAC-3' for the reverse primer, which were designed to amplify from ‒145 bp to ‒8 bp of the *IRF6* gene based on the major *IRF6* start site. The PCR products were cloned into a cloning vector, and 12 clones were cycle-sequenced for each sample.

### Quantitative real-time reverse transcription-PCR (RT-PCR)

Total RNA was treated with DNase I (Ambion, Austin, TX) and cDNA was synthesized from 1.0 μg of total RNA using a Superscript III kit (Life Technologies, Rockville, MD). RT-PCR was performed using the SYBR Green PCR Master Mix I (Toyobo, Osaka, Japan) and the 7500 Real-Time PCR System (Applied Biosystems, Foster City, CA). The sequences of the primers were: 5'-CCGTTTGAGATCTACTTATGCT-3' for the forward primer and 5'-GATCATCCGAGCCACTACTG-3' for the reverse primer. The number of molecules of a specific gene in the sample was measured by comparing its amplification with that of standard samples, which contained 10^1^ to 10^8^ copies of the gene. The mRNA quantity of each gene was normalized with that of the glyceraldehyde-3-phosphate dehydrogenase gene (*GAPDH*).

### Statistical analysis

Statistical analysis was performed using the commercially available software, SPSS version 18 (SPSS Japan, Tokyo, Japan). Significant differences in laboratory data were analyzed by linear regression analysis, the Kruskal-Wallis test, the Mann-Whitney U test and the Log-rank test. *P* < 0.05 was considered statistically significant.

## Results

### The methylation level of the 5' *IRF6* CGI is completely inversely correlated with melanoma cell sensitivity to IFN-β

**Fig 1:**
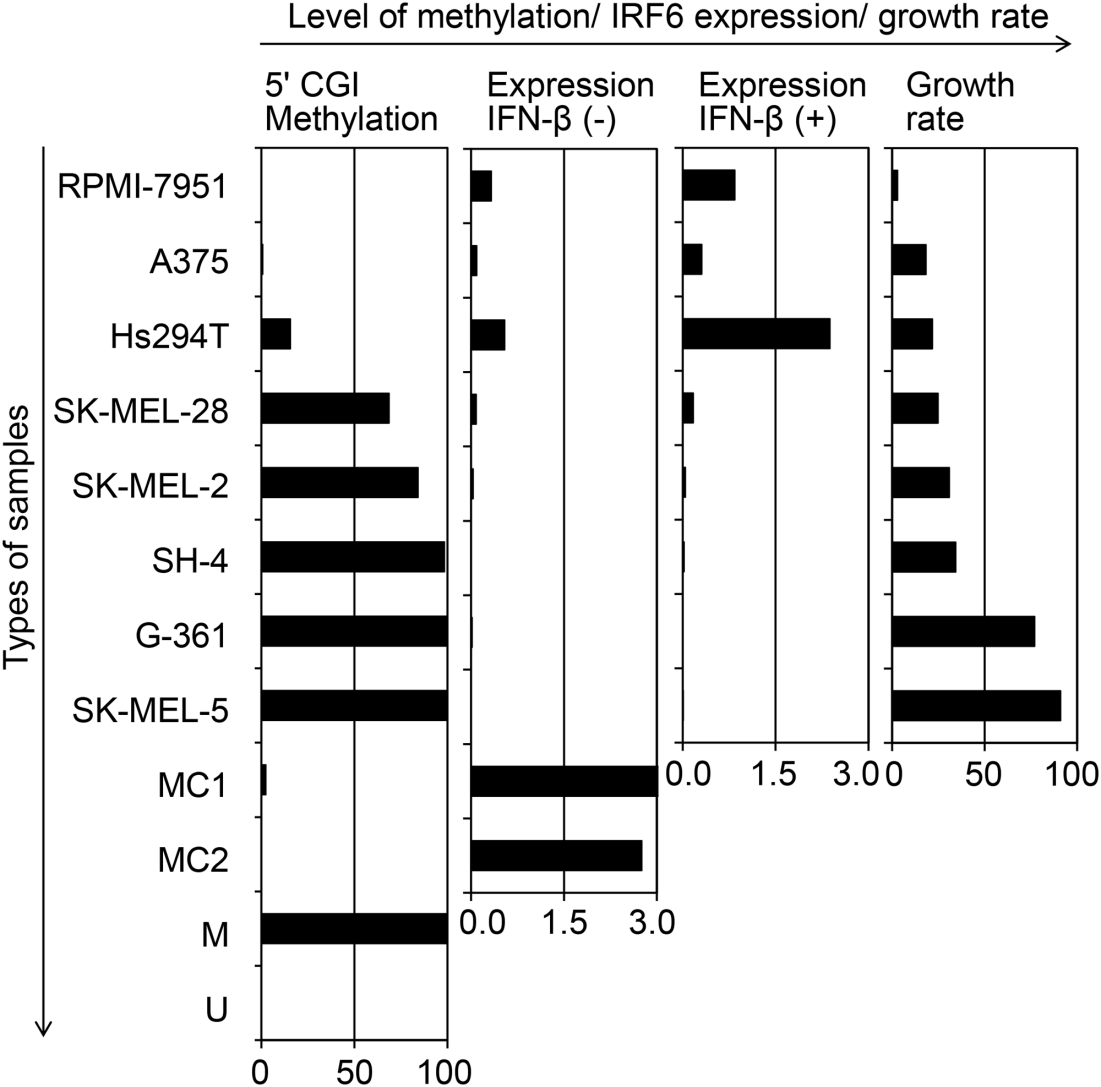
Concordance of the methylation status of the 5' *IRF6* CGI with *IRF6* transcription or IFN-β sensitivity in melanocyte-lineage cells. The left bar graph indicates the methylation levels (%) of the 5' *IRF6* CGI in the indicated melanoma cell lines, normal cultured melanocytes (MC1 and MC2) and controls including methylated DNA (M) and unmethylated DNA (U). The second bar graph from the left indicates the expression levels of *IRF6* divided by the expression levels of *GAPDH* (×10^−4^) in each of the melanoma cell lines and normal cultured melanocytes (MC1 and MC2) that were not treated with IFN-β. The expression level of MC1 was 1.1×10^−3^, which is higher than the 3.0×10^−4^ upper limit of the graph. The third bar graph from the left indicates the expression levels of *IRF6* divided by the expression levels of *GAPDH* (×10^−4^) in each of the melanoma cell lines treated with IFN-β. The fourth bar graph from the left indicates the cell growth rate which was defined as the value of the number of IFN-β-treated cells divided by the number of PBS-treated cells. All four bar graphs correspond to each other with respect to the melanoma cell lines and normal cultured melanocytes.

The methylation status of the 5' *IRF6* CGI in melanoma cell lines was analyzed using methylation-specific PCR, and the sensitivity of the same melanoma cell lines to IFN-β was analyzed using cell growth assay (Fig 1). Statistical analysis revealed a significant correlation between 5' *IRF6* CGI methylation levels and sensitivity to IFN-β (linear regression analysis, *P* = 0.033). Thus, the methylation level of the 5' *IRF6* CGI was completely inversely correlated with IFN-β sensitivity in melanoma cell lines.

### The methylation status of the 5' *IRF6* CGI regulates the expression of *IRF6*

**Fig 2:**
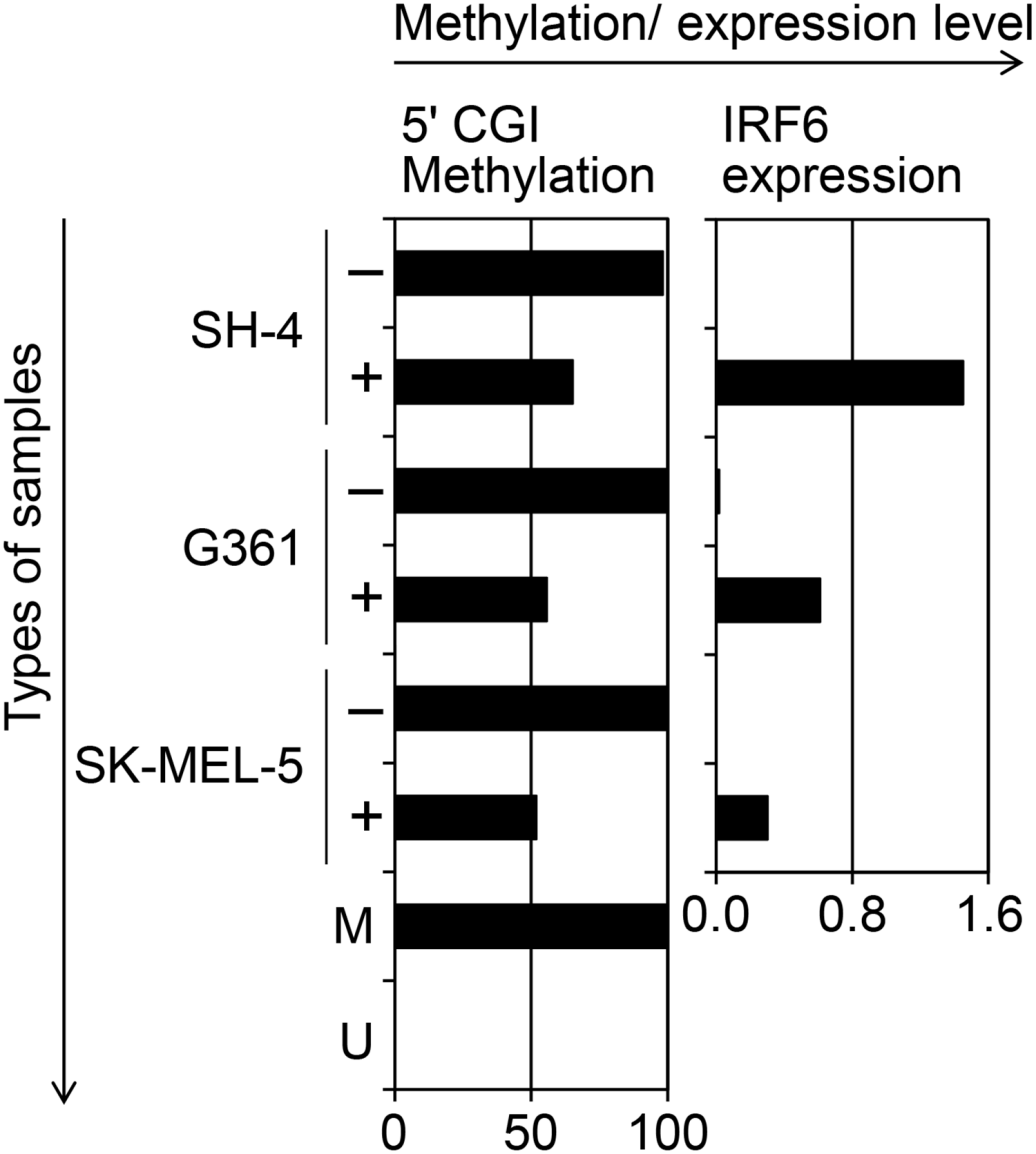
Transcriptional induction of *IRF6* in SH-4, G-361 and SK-MEL-5 cells after treatment with the demethylating agent 5-aza-dC. The left bar graph indicates the methylation levels (%) of the 5' *IRF6* CGI and the right bar graph indicates the expression levels of *IRF6* divided by those of *GAPDH* (×10^−4^), in SH-4, G-361 and SK-MEL-5 cells that were treated with the demethylating agent (+) or with PBS (-). Both bar graphs correspond to each other with respect to the cell lines.

The association between the methylation status of the 5' *IRF6* CGI (S1 Fig) and transcription of *IRF6* as analyzed by RT-MSP and RT-PCR, respectively (Fig 1), for each of the melanoma cell lines (RPMI-7951, A375, Hs294T, SK-MEL-28, SK-MEL-2, SK-MEL-5, SH-4 and G-361) and normal cultured melanocytes (MC1 and MC2) was as follows. Some of the cell lines with expression of *IRF6*, including the RPMI-7951, Hs294T, and SK-MEL-2 cell lines and normal cultured melanocytes (MC1 and MC2), exhibited a considerable amount of unmethylated DNA molecules (methylation levels ranging from 0.0% to 53.3%). Other cell lines with high methylations levels (ranging from 87.0% to 97.2%), including A375, SK-MEL-5, SH-4 and G-361 cells, exhibited suppression of *IRF6* expression (Fig 1). Treatment with the demethylating agent 5-aza-dC resulted in transcriptional induction of *IRF6* in SH-4, G-361 and SK-MEL-5 cells, which had exhibited high methylation levels and suppression of *IRF6* before this treatment (Fig 2).

### IFN-β enhances *IRF6* expression in unmethylated melanoma cells

We then determined the association between IFN-β treatment and *IRF6* expression in the individual melanoma cell lines according to their 5' *IRF6* CGI methylation statuses as shown in Fig 1. IFN-β enhanced *IRF6* expression in the melanoma cell lines in which there was a considerable amount of unmethylated 5' *IRF6* CGI DNA molecules but had a lower effect on *IRF6* expression in melanoma cell lines that had only a small amount of unmethylated DNA molecules.

### The 5' *IRF6* CGI is methylated *in vivo* in melanoma but not in melanocytic nevus

**Fig 3:**
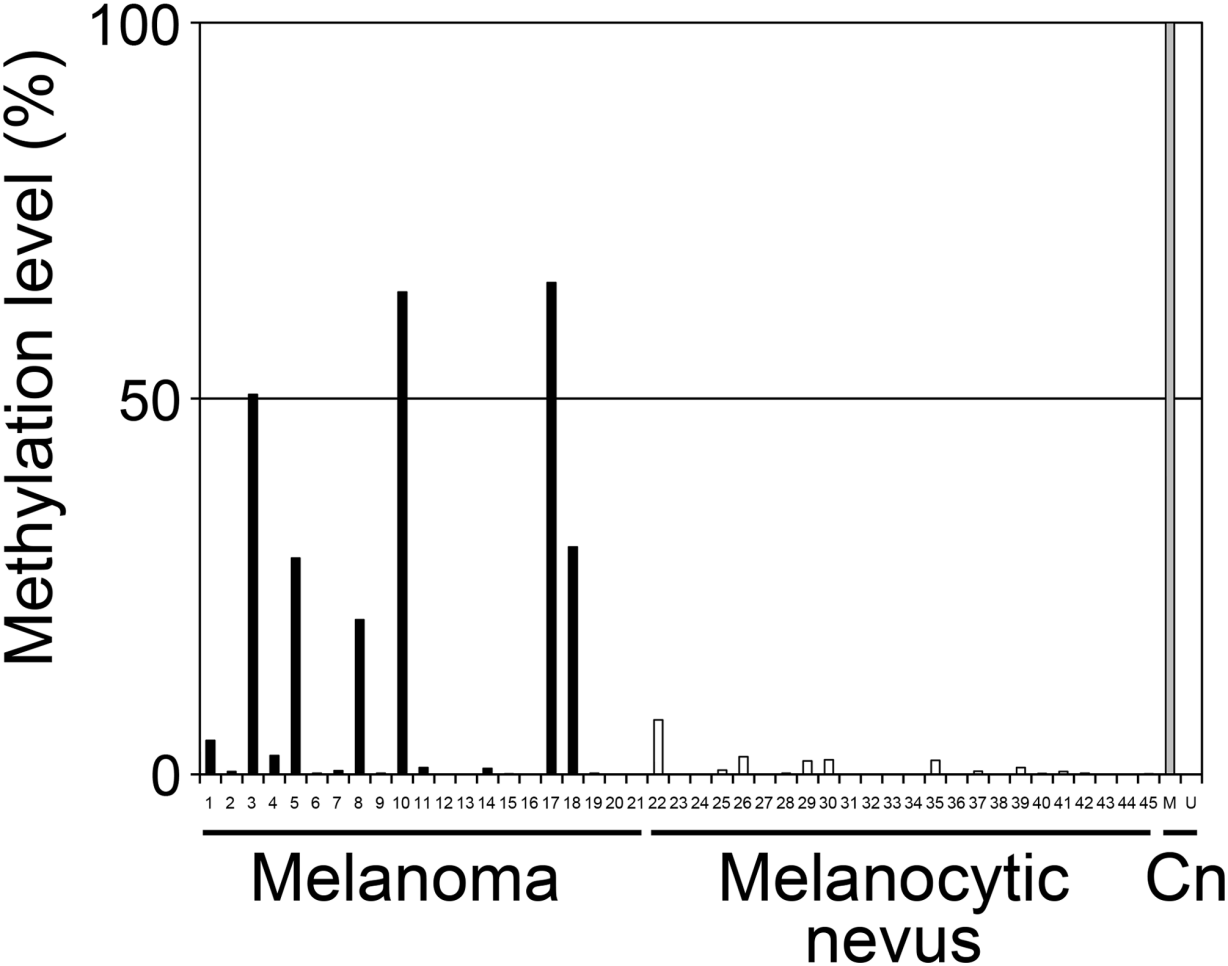
Methylation levels of the CGI located in the 5' region of *IRF6* in 21 clinical melanoma samples and 24 clinical melanocytic nevus samples. Each methylation level was determined using RT-MSP. The vertical and horizontal axes indicate the methylation level (%) and sample ID, respectively. M and U indicate controls of methylated and unmethylated DNA, respectively.

The *in vivo* methylation status of *IRF6* in melanomas and melanocytic nevi was analyzed by performing RT-MSP of 21 clinical melanoma samples and 24 clinical melanocytic nevus samples. The methylation levels ranged widely from 0.0% to 65.4% in clinical melanoma samples including sample #17 with a methylation level of 65.4% and sample #10 with a level of 64.2%, while there was a narrow range of low methylation levels, ranging from 0.0% to 7.2%, in clinical melanocytic nevus samples (Tables 1 and 2 and Fig 3). Consequently, six out of 21 melanoma samples (28.6%) showed higher methylation level than the highest methylation level in the melanocytic nevus samples.

### Data from bisulfite sequencing are compatible with those from RT-MSP

**Fig 4:**
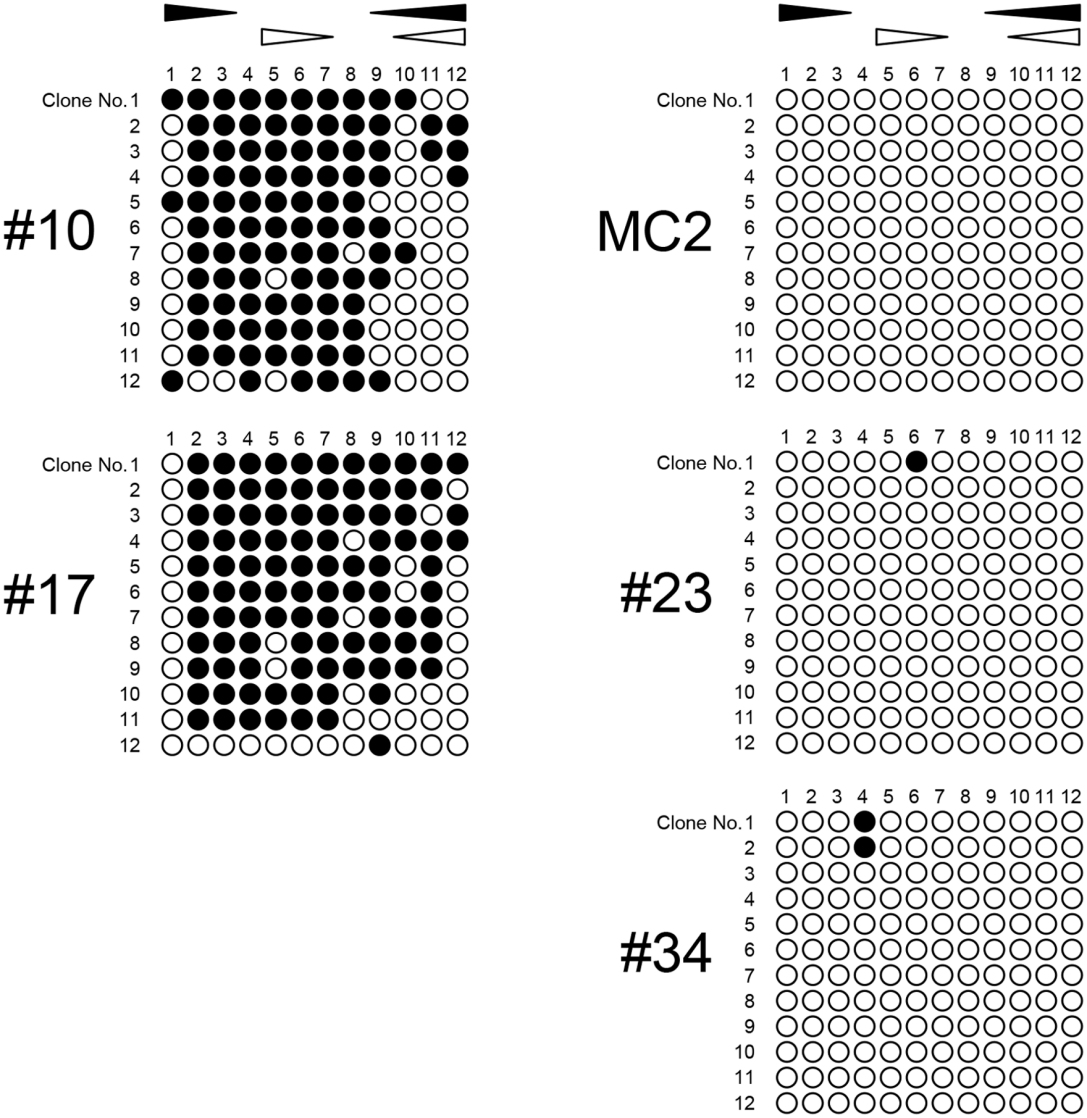
Bisulfite sequencing analysis to confirm the RT-MSP data. The results of bisulfite sequencing of the 5' *IRF6* CGI for representative samples are shown. The clinical melanoma samples #10 and #17 were examined as representatives of samples with high methylation levels. Normal cultured melanocytes MC2 and clinical melanocytic nevus samples #23 and #34 were examined as representatives of samples with low methylation levels. Closed and open circles indicate methylated and unmethylated CpG sites, respectively. Closed and open triangles indicate the location of the RT-MSP primer sets specific to the methylated and unmethylated DNA sequences, respectively. The vertical and horizontal numbered rows indicate each clone and CpG site, respectively.

The data obtained using RT-MSP were confirmed by bisulfite sequencing using samples with representative RT-MSP results. For this analysis, clinical melanoma samples #10 and #17 were examined as representatives of samples with high methylation levels. Normal cultured melanocytes MC2 and clinical melanocytic nevus samples #23 and #34 were examined as representatives of samples with low methylation levels (Fig 4). The data showed that CpG sites within the 5' *IRF6* CGI were densely methylated in samples #10 and #17, while they were sparsely methylated in MC2 cells and in sample #23 and #34. Thus, the bisulfite sequencing data were compatible with the results of RT-MSP.

### The methylation level of the 5' *IRF6* CGI is associated with melanoma subtype

The correlations between the methylation status of the 5' *IRF6* CGI and clinical parameters such as age, sex, subtype and outcome were analyzed by statistical analyses (Table 3). The Kruskal-Wallis test indicated a significantly different distribution of the methylation levels of the 5' *IRF6* CGI among the subtypes of melanoma (*P* = 0.035); the methylation levels of the acral type tended to be higher than those of the other types. On the other hand, Kaplan-Meier survival analysis and the log-rank test indicated no significant difference in outcome between the patients who donated melanoma samples whether the samples were with or without methylated DNA molecules. In this analysis, the cut-off value for methylation was set at 7.2%, which was the highest methylation level of the 24 melanocytic nevus samples. Similarly, the clinical parameters tested, including age and sex, were not associated with methylation levels of the 5' *IRF6* CGI.

**Table 3:** Correlation between tumor methylation levels and clinical parameters.

Parameters	*P*-value	Statistical analysis method
Age	0.304	Linear regression analysis
Sex	0.445	Mann-Whitney U test
T-classification	0.348	Kruskal-Wallis test
N-classification	0.655	Kruskal-Wallis test
M-classification	0.462	Mann-Whitney U test
Type	0.035	Kruskal-Wallis test
Outcome*	0.719	Log-rank test

*The cut off value of the methylation level was 7.2% which was the highest value of the methylation levels in the melanocytic nevus samples.

## Discussion

The present study indicated for the first time that (i) the 5' *IRF6* CGI is sometimes methylated, resulting in silencing of *IRF6*, in melanoma but not in melanocytic nevus, and (ii) the methylation status of the 5' *IRF6* CGI is potentially associated with the sensitivity of melanoma to IFN-β.

The *in vitro* examinations showed that (i) some melanoma cell lines with high methylation levels of the 5' *IRF6* CGI exhibited suppression of the gene, (ii) other melanoma cell lines with low methylation levels of this CGI exhibited expression of the gene, and (iii) treatment with a demethylating agent restored *IRF6* gene expression in the melanoma cell lines that had high methylation levels and suppression of *IRF6* before the treatment. These data indicated that (i) the methylation status of the analyzed region within the CGI located in the 5' region of *IRF6* regulates the expression of the gene, and (ii) *IRF6* can be silenced through methylation of the 5' *IRF6* CGI in melanoma. These *in vitro* data encouraged us to investigate the *in vivo* methylation status of *IRF6* in melanoma.

The *in vivo* examinations indicated that the clinical melanocytic nevus samples exhibited a narrow and range of low methylation levels, which ranged from 0.0% to 7.2%, and that the clinical melanoma samples exhibited a wide range of methylation levels, which ranged from 0.0% to 65.4% and included a number of samples with high methylation (e.g. 65.4% methylation in sample #17 and 64.2% in #10). Six of the 21 clinical melanoma samples exhibited a methylation level that was higher than the highest methylation level of the clinical melanocytic nevus samples. These results suggested that a certain percentage of melanomas may develop in association with the silencing of *IRF6* unlike nevus melanocytic.

The present study also demonstrated that (i) the methylation level of the 5' *IRF6* CGI was completely inversely correlated with the sensitivity to IFN-β of the eight melanoma cell lines examined, and (ii) IFN-β enhanced *IRF6* expression in the melanoma cell lines that had a considerable amount of unmethylated 5' IRF6 CGI DNA molecules. These observations strongly suggested that cell sensitivity to IFN-β is dependent on the methylation status of the 5' *IRF6* CGI, which regulates the *IRF6* gene expression level. This consideration led to the hypotheses that (i) IFN-β treatment enhanced *IRF6* expression in the melanoma cells with an unmethylated 5' *IRF6* CGI, with the result that IRF6-associated tumor suppressors, including maspin and IRF6-induced endogenous IFNs, inhibit the growth of melanoma cells with an unmethylated 5' *IRF6* CGI, and (ii) IFN-β treatment cannot induce IRF6 expression in the melanoma cells with methylated 5' *IRF6* CGI, with the result that IRF6-associated tumor suppressors do not inhibit the growth of melanoma cells with a methylated 5' *IRF6* CGI. Therefore, methylation analysis of the 5' *IRF6* CGI may be useful for the selection of IFN-β treatment-sensitive melanomas.

The *in vitro* data indicated that the melanoma cell lines with suppression of *IRF6* exhibited a methylation level >68.5%, while the *in vivo* data indicated that the highest value of such methylation levels among melanocytic nevus samples was 7.2%, and that the methylation levels of 21 clinical melanoma samples ranged from 0.0% to 65.4%. The clinical melanoma samples included a considerable amount of tumor-surrounding tissues with normally low methylation levels. Therefore, clinical samples with a methylation level >7.2% should be regarded as having a highly methylated 5' *IRF6* CGI, even though their methylation levels are ≤68.5%.

Statistical analyses indicated a significantly different distribution of the methylation levels of the 5' *IRF6* CGI among the subtypes of melanoma (Kruskal-Wallis test, *P* = 0.035); the acral type tended to be higher than those of the other types. These results suggested that (i) Curtin's classification of melanoma may be associated with epigenetic abnormalities as well as genetic abnormalities [25], and (ii) IFN-β-resistant melanomas are more frequent in acral melanomas than in the other types of melanomas.

Bisulfite sequencing was performed to confirm the results of RT-MSP. The data were generally consistent with those of RT-MSP. This examination indicated that the CpG sites within the 5' *IRF6* CGI were densely methylated in the clinical melanoma samples in which high methylation levels were detected by RT-MSP (e.g., sample #10 with a 64.2% methylation level and sample #17 with a 65.4% level). Conversely, this examination indicated that these CpG sites were sparsely methylated in the samples in which low methylation levels were detected by RT-MSP (e.g., MC2 cells with a 0.1% methylation level and samples #23 and #34 with a 0.0% level). These data suggested that RT-MSP is a reliable and reasonable procedure to use for determination of the methylation status of the 5' *IRF6* CGI.

One limitation of this study is that only 21 advanced stage melanoma samples were analyzed. Our data derived from the statistical analyses should be confirmed in a larger number of melanoma samples. Another limitation of this study is that data regarding the IRF6 protein level were not provided. The association between the methylation status of the 5' *IRF6* CGI and IRF6 protein expression should be concurrently evaluated.

In conclusion, *IRF6* is aberrantly silenced due to DNA methylation of the CGI located in the 5' region of the *IRF6* gene in melanomas, but not in melanocytic nevi. The methylation levels of 5' *IRF6* CGI are potentially associated with the sensitivity of melanoma cells to IFN.

## Supporting information

S1 Fig Gene structure of the 5' region of *IRF6*. The gray square indicates exon 1. The long vertical line indicates the transcriptional start site. Vertical short lines show individual CpG sites (top) and GpC sites (bottom). The regions analyzed using the methylated DNA-specific primer set and the unmethylated-DNA sequence-specific primer set in RT-MSP are shown as a black and a gray horizontal line, respectively. The region analyzed by bisulfite sequencing is shown as a double horizontal line.
(PDF)
