# Supplementary figures and images for: Silencing of interferon regulatory factor gene 6 in melanoma
Source: PLoS One. 2017 Sep 6;12(9):e0184444. doi: 10.1371/journal.pone.0184444 (PMC5587289; doi:10.1371/journal.pone.0184444)

# S1 Fig

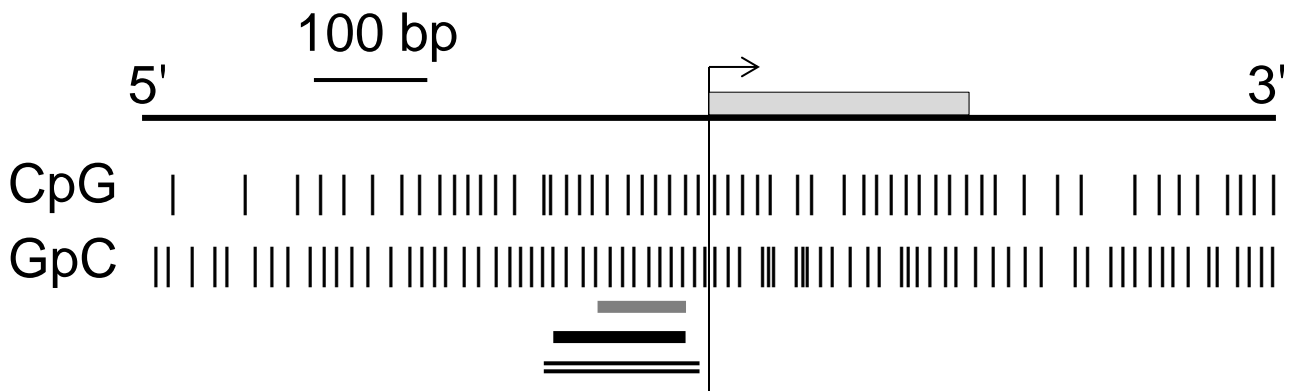

Supplement: S1 Fig — The gray square indicates exon 1. The long vertical line indicates the transcriptional start site. Vertical short lines show individual CpG sites (top) and GpC sites (bottom). The regions analyzed using the methylated DNA-specific primer set and the unmethylated-DNA sequence-specific primer set in RT-MSP are shown as a black and a gray horizontal line, respectively. The region analyzed by bisulfite sequencing is shown as a double horizontal line. (PDF) [file pone.0184444.s001.pdf]
